# The FACE test: a new neuropsychological task to assess the recognition of complex mental states from faces

**DOI:** 10.1007/s10072-023-06697-w

**Published:** 2023-02-28

**Authors:** Stefano Terruzzi, Giulia Funghi, Claudia Meli, Nicole Barozzi, Francesca Zappini, Costanza Papagno, Alessandra Dodich

**Affiliations:** 1 Center for Mind/Brain Sciences-CIMeC, University of Trento, Rovereto, Italy; 2 Department of Psychology, University of Milano-Bicocca, Milan, Italy

**Keywords:** Social cognition, Mental state recognition, Facial expressions, Parkinson’s disease

## Abstract

**Background:**

Social cognition deficits are reported in several neurodegenerative diseases, including Parkinson’s disease (PD). However, the availability of tasks for the clinical assessment is still limited, preventing the full characterization of socio-cognitive dysfunctions in neurological patients. This study aims to present a new task to assess the recognition of complex mental states from faces (FACE test), reporting normative data for the Italian population and an example of its clinical application to 40 PD patients.

**Methods:**

Two-hundred twenty-nine Italian participants with at least 5 years of education were enrolled. Data were analyzed according to the method of equivalent scores; test-retest reliability and convergent validity were assessed. Two short versions of the FACE test were defined for clinical and research purposes. The prevalence of deficits in the FACE test was computed in the PD sample, as well as correlations with cognitive performance and diagnostic accuracy.

**Results:**

Regression analyses revealed significant effects of demographic variables on FACE performance, with younger and more educated individuals showing higher scores. Twenty-eight percent of PD patients showed borderline/pathological performance, which was correlated with emotion recognition/attribution abilities, and attentive-executive functions. The FACE test was accurate (80%) in distinguishing PD patients with socio-cognitive dysfunctions from both controls and PD patients without emotion recognition/attribution difficulties.

**Conclusion:**

The FACE test represents a new tool assessing the ability to recognize complex mental states from facial expressions. Overall, these results support its use in both clinical and research settings, as well as the presence of affective processing deficits in a subsample of PD patients.

**Supplementary Information:**

The online version contains supplementary material available at 10.1007/s10072-023-06697-w.

## Introduction

Social cognition refers to a broad range of cognitive abilities which allow humans to understand and interact with others by adopting appropriate, goal-directed, behaviors [1]. As a multidimensional construct, it involves different subdomains, usually referred to as social perception, theory of mind (ToM), empathy, and social behavior [2]. Social perception includes the ability to distinguish others by facial features, body postures, or voices, and to recognize their identity, as well as their emotions, from these features [3]. ToM refers to the ability to infer others’ complex mental states such as thoughts, intentions, beliefs, and motivations (cognitive ToM: “I understand what you think”), as well as feelings and emotional states (affective ToM: “I understand what you feel”) [4]. Together with empathy, identified as the ability to represent, share, and predict what another person is feeling [5], these social cognitive skills are crucial to predicting others’ behavior and guiding adaptive and homeostatic behavioral responses.

Social cognition has been recently included as a stand-alone cognitive domain in the American Psychiatric Association’s Diagnostic and Statistical Manual for Mental Disorders — DSM-5 [6], recognizing that socially inappropriate behavior can represent a clinical symptom in several neurodevelopmental, neuropsychiatric and neurodegenerative disorders. Social cognitive deficits represent a core cognitive feature from the early stages of the behavioral variant of frontotemporal dementia (bvFTD) [7] and might characterize different clinical conditions [8], among which are other syndromes of the FTD spectrum, as well as amyotrophic lateral sclerosis, Alzheimer’s disease, and atypical parkinsonisms [9 for review].

Among the different neurodegenerative conditions associated with cognitive decline, increasing evidence points to the presence of social cognitive dysfunctions in Parkinson’s disease (PD). Indeed, a meta-analytic review has revealed significant deficits of emotion recognition [10], at least partially independent of patients’ level of motor disability, depressive symptoms, executive and visual processing impairment, and disease duration and severity [10, 11]. This disorder seems particularly pronounced for negative emotions, although contrasting results have been reported [10]. Together with emotion recognition dysfunctions, deficits of both ToM subcomponents have been described across the entire PD disease course [12, 13]. Initial evidence pointed towards a greater impairment of cognitive aspects of ToM [14], calling into question the spatiotemporal progression of striatal dopamine reduction in PD first involving regions associated with cognitive ToM (e.g., dorsolateral prefrontal cortex and striatum) [4]. However, recent evidence underlines an early alteration also in emotion attribution in these patients [12], and deficits in the representation of affective mental states have been now recursively reported (see Enrici et al. [15] for a review of the main findings).

The clinical relevance of these deficits in PD has recently been further emphasized by preliminary evidence suggesting socio-emotional impairments at the clinical neuropsychological assessment in 20 to 30% of PD patients, even in the early stages of the disease [16, 17].

Notably, despite the extensive body of literature supporting the possible presence of socio-cognitive dysfunctions, its clinical assessment is still limited due to the paucity of clinical tools available [18, 19], as shown in Table S1 of Supplementary Information, reporting the list of social cognition tests adapted in Italian for clinical use. There is thus an urgent need to develop and introduce new standardized tasks in the clinical practice to fully characterize socio-cognitive dysfunctions in neurodegenerative populations.

The aim of this study is to present a new test assessing complex mental state recognition through faces (FAcial Complex Expressions (FACE) test) and to provide normative data for the Italian population. In addition, the FACE test has been tested on a sample of 40 PD patients, as a demonstration of its clinical application in a disorder possibly characterized by social cognition impairments. The prevalence of deficits in complex mental state recognition was computed in the PD sample, as well as the association with performance in various cognitive domains (i.e., learning and memory, language, complex attention, executive functions, and perceptual-motor abilities), including social cognition, under the hypothesis of a specific correlation between the FACE test and socio-cognitive task performance. Finally, to provide disease-specific cutoff scores, receiver operating characteristic (ROC) analyses were performed comparing PD patients characterized by difficulties in emotion recognition/attribution detected by other socio-cognitive tasks with unimpaired PD patients and healthy controls.

## Materials and methods

### Participants

A sample of 229 healthy adult Italian-native speakers (137 women; mean age = 53.3 ± 18.6 years; mean education = 13.3 ± 4.2 years; mean Montreal Cognitive Assessment [MoCA] raw score = 26.2 ± 2.8) was voluntarily recruited for the normative procedure. Exclusion criteria were a history or clinical evidence of neurological or neuropsychiatric diseases and a MoCA adjusted score below the national cutoff score [20]. Sample stratification for age, education, and sex is reported in Table 1.

**Table 1:** Stratification for age, education, and sex of the normative sample

Age in years									
Education in years	18–29	30–39	40–49	50–59	60–69	70–79	≥80	Total (F/M)	Total
≤5	NA	NA	NA	NA	1/0	3/1	6/1	10/2	12
6–8	NA	NA	2/1	8/4	6/5	7/3	1/1	24/14	38
9–16	14/6	7/9	11/7	15/8	13/9	6/6	3/3	69/48	117
≥17	7/7	6/6	6/4	6/2	4/5	4/3	1/1	34/28	62
Total (F/M)	21/13	13/15	19/12	29/14	24/19	20/13	11/6	137/92	229
Total	34	28	31	43	43	33	17	229	**-**

In each cell, the number of participants as females/males is reported. *F* female, *M* male, *NA* not available

A group of 40 PD patients (15 women; mean age = 70.4 ± 5.3 years; mean education = 11.4 ± 4.0 years; mean Montreal Cognitive Assessment [MoCA] raw score = 22.7 ± 3.8), diagnosed according to the United Kingdom Parkinson’s Disease Society brain bank criteria [21], was recruited at the Center for Neurocognitive Rehabilitation of the Center for Mind/Brain Sciences (University of Trento). Inclusion criteria were (i) a diagnosis of idiopathic PD; (ii) a Hoehn and Yahr score ≤3 [22]; (iii) being under anti-parkinsonian medication; and (iv) age above 60 years old. Patients with dementia or other neuropsychiatric disorders were excluded. A baseline clinical and neuropsychological assessment, including socio-cognitive evaluation, was performed by experienced neurologists and neuropsychologists, and patients were tested while in their medication-on condition. All participants provided informed consent to the study, which was conducted following the ethical guidelines of the local ethics committee and the Declaration of Helsinki.

### FACE test

The FACE test is a recognition task of complex mental states from facial expressions. It consists of 36 high-quality pictures derived from the McGill Face Database [23], which contains high-resolution validated pictures of 93 expressions of mental states interpreted by two professional actors (1 male and 1 female) in front and side views. Details on test construction are reported in Supplementary Information. Briefly, we performed a pilot study on 30 young healthy participants to select those images from the McGill Face Database judged to be the most representative of the different mental states. Participants were asked to judge the valence and arousal of the presented pictures through a Likert-point system, as well as to select the best definition of mental states based on three different options, thus creating a glossary that could be used during administration of the task.

**Fig. 1:**
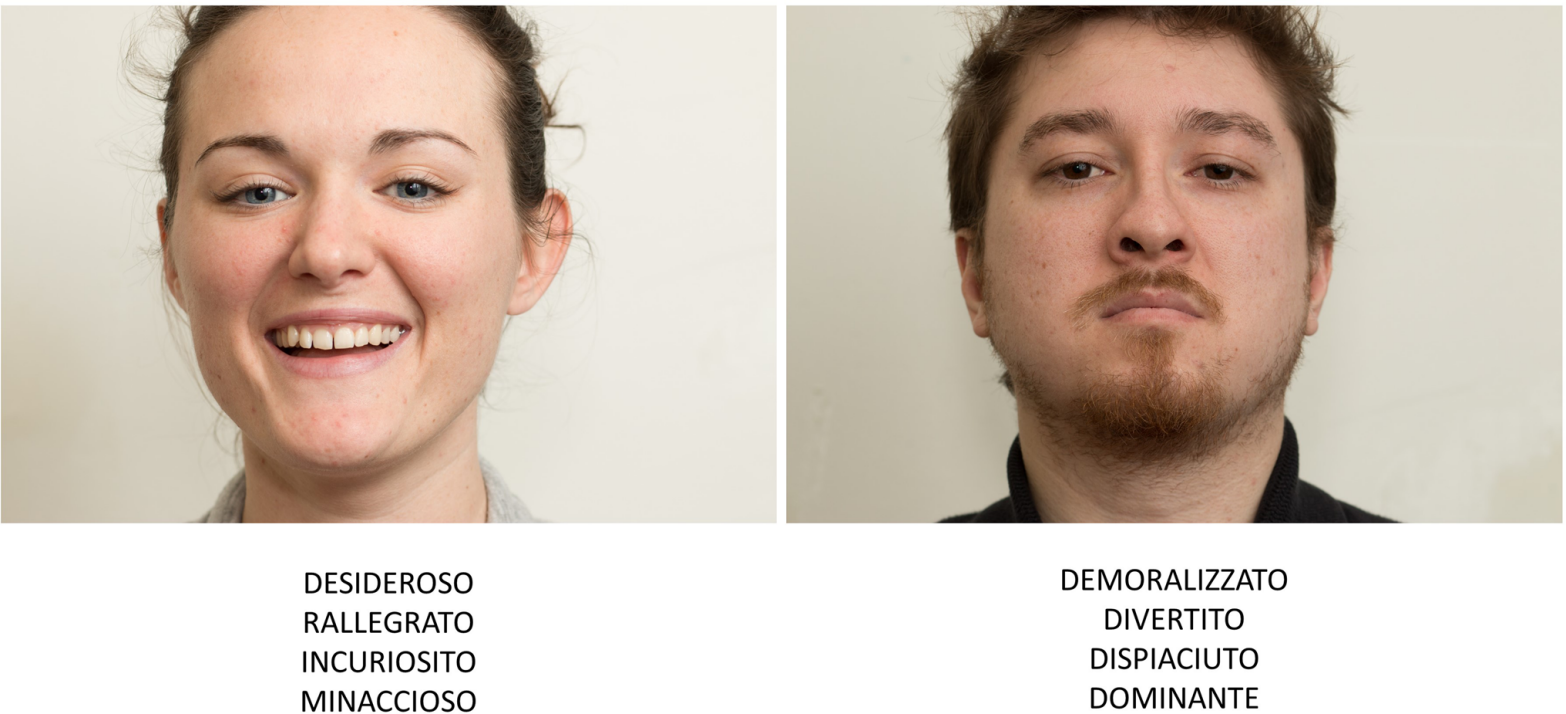
Examples of images from the FACE test. Translation from Italian to English of examples of complex mental state labels: Desideroso = Desire; Rallegrato = Amused; Incuriosito = Intrigued; Minaccioso = Threatening; Demoralizzato = Dispirited; Divertito = Entertained; Dispiaciuto = Apologetic; Dominante = Dominant

Based on the results of the pilot study, the final version of the test consisted of 36 stimuli (see Supplementary Information, Table S2). Each stimulus was presented centrally on the screen, together with four complex mental state labels, of which one represented the target response and the other three, incorrect alternatives (see Fig. 1). Participants were required to answer verbally, choosing the label that best described the actor’s facial expression; no time limits were applied. Participants were also given the glossary and were encouraged to consult it for any label’s meaning they were unsure of. A trial run preceded the task, consisting of an example of a stimulus that would not appear in the test, with associated response alternatives. The score range was 0–36, with a higher score corresponding to a better performance. For both clinical and research purposes requiring multiple administrations of the task, two 18-item versions of the FACE test (i.e., FACE — Version A and FACE Version B) were derived from the 36-item version (see Supplementary Information for details on the construction of the short forms).

### Statistical analyses

For the 36-item FACE test score, descriptive statistics were computed based on the performance of the 229 healthy control participants. Preliminary normality checks on raw scores were performed by both evaluating data distribution (Shapiro-Wilk test), and visually inspecting plots for outlier detection. Due to non-normal distribution (age: Shapiro-Wilk *W* = 0.965, *p* < 0.001; education: Shapiro-Wilk *W* = 0.965, *p* < 0.001; FACE test: Shapiro-Wilk *W* = 0.962, *p* < 0.001), between-variable associations (age, years of education, and FACE test raw score) were verified through non-parametric correlation analysis (Spearman’s rank correlation coefficient, *r*_s_). Sex differences were tested via the Mann-Whitney test (independent sample).

Regression-based norms, as well as the equivalent scores (ES), were derived according to the standardization system proposed by Capitani and Laiacona [24]. In detail, different linear regression analyses were performed to establish which demographic variables, including age and years of education (including their quadratic, logarithmic, inverse, and square-root terms), had to be included in the final model as the most effective in reducing the residual variance. Adjusted values were computed by adding or subtracting the contribution of each demographic variable for each subject. The correction grid was then derived to adjust the performance of each newly tested subject for the effect of the demographic variables. Subsequently, the adjusted scores were classified into five categories, the equivalent scores (ES), ranging from 0 to 4. In particular, the “0” corresponds to the adjusted score equal to or lower than the outer unidirectional non-parametric tolerance limit, with a confidence of 95% (the sixth observation for 229 subjects). The “4” category corresponds to scores higher than the median value of adjusted scores. Equivalent scores “1,” “2,” and “3” are intermediate values on a quasi-interval scale calculated with reference to the left half of the distribution.

Intraclass correlation coefficient (ICC) and Spearman’s rank correlation coefficient were used to evaluate, respectively, test-retest reliability and convergent validity between the FACE test performance and the Ekman 60-Faces Test [25] in a subsample of 30 healthy participants.

The equivalence of the two 18-item short forms was preliminarily assessed in healthy participants following previous studies [26] and through a two-one-sided test procedure (TOST) for dependent sample, performed via the R package TOSTER (https://cran.r-project.org/web/packages/TOSTER/TOSTER.pdf) [27]. The upper and lower equivalence bounds were defined as −0.5 and +0.5, respectively, and *α* level = 0.05. The raw scores obtained in the short forms were then transformed into a 36-point raw score, on which adjustment based on normative data can be applied to derive the ES (see Supplementary Information for details on the conversion procedure).

To test the use of the FACE test in clinical practice, we applied the normative procedure to the performance of a sample of 40 PD patients, computing the prevalence of socio-cognitive deficits in complex mental state representation. Moreover, to explore the relationship between FACE test performance and cognitive functioning we computed Spearman’s rank partial correlation, controlling for MoCA raw score, between FACE test raw scores and variables representing cognitive functions in different domains (working memory, long-term memory, executive functions, visuospatial abilities, semantic access), as well as in social cognition subdomains (i.e., emotion recognition — Ekman 60-Faces Test [25], cognitive and affective TOM — Story-based Empathy Task [28]). The *p*-values resulting from correlation analyses were corrected for multiple comparisons using Bonferroni’s method (*α*_adjusted_ = 0.05/*k*, with *k* equal to the number of correlations performed, i.e., *k* = 16).

Finally, FACE test diagnostic accuracy was tested via ROC curve analysis, comparing PD patients (*n* = 14) showing difficulties at social tasks of emotion recognition and/or attribution (ES equal to 0 or 1) with the normative sample (*n* = 229) and with unimpaired PD patients (*n* = 26). All statistical analyses were performed using Jamovi 2 [29].

## Results

FACE test descriptive data of the 229 healthy controls are reported in Table 2. Correlation analyses revealed a negative association between FACE test raw scores and age (*r*_s_ = −0.502, *p* < 0.001), and a positive correlation with years of education (*r*_s_ = 0.441, *p* < 0.001). Independent sample *t*-tests to verify the impact of sex on FACE performance did not show significant differences (*U* = 6244, *p* = 0.921).

**Table 2:** FACE test descriptive data of 229 healthy controls

Education in years	Age in years						
	18–29	30–39	40–49	50–59	60–69	70–79	≥80
≤5	NA	NA	NA	NA	21.0	27.8 ± 6.4	23.6 ± 2.9
6–8	NA	NA	26.0 ± 1.0	27.8 ± 3.0	26.9 ± 2.9	26.7 ± 3.5	28.0 ± 0.0
9–16	30.6 ± 3.1	31.8 ± 2.2	30.1 ± 2.8	29.1 ± 2.9	28.1 ± 3.6	26.1 ± 3.5	25.8 ± 2.0
≥17	32.3 ± 2.8	31.7 ± 2.9	31.0 ± 2.6	30.4 ± 1.5	28.2 ± 3.0	29.4 ± 2.9	30.0 ± 4.2

In each cell, mean ± standard deviation is reported

The final model of multiple regression showed age and the square root of education in years as the best predictors of FACE performance (*F*_(2,226)_ = 53.0, *p* < 0.001; adjusted *R*^2^ = 0.313, Breusch-Pagan *p* = 0.136), with higher scores for younger and more-educated subjects. Table 3 reports the adjustment grid with the correction factors to be added or subtracted from FACE test raw scores. ES and intervals for the FACE test global score are shown in Table 4.

**Table 3:** Age and education adjustment grid for FACE test score

Education in years	Age in years													
	20	25	30	35	40	45	50	55	60	65	70	75	80	85
5									2.73	3.09	3.45	3.81	4.17	4.54
8					0.30	0.67	1.03	1.39	1.75	2.11	2.47	2.84	3.20	3.56
13	−2.42	−2.06	−1.70	−1.34	−0.98	−0.62	−0.25	0.11	0.47	0.83	1.19	1.55	1.91	2.28
17	−3.28	−2.92	−2.55	−2.19	−1.83	−1.47	−1.11	−0.75	−0.38	−0.02	0.34	0.70	1.06	1.42

Adjusted FACE test score = raw score + 0.0723 × (Age − 53.275) − 1.6493 × (√Education − 3.595)

**Table 4:** Equivalent scores and intervals for the FACE test

Equivalent scores	Adjusted score intervals
0	≤22.685
1	22.686–24.611
2	24.612–27.159
3	27.160–29.491
4	≥29.492

Outer tolerance limit: 22.685, inner tolerance limit: 24.244

Spearman’s rank correlation coefficient showed good convergent validity (*r*_s_ = 0.578, *p* < 0.001); test-retest reliability was moderate (ICC = 0.66). When comparing the two 18-item short forms, no significant differences were found (*t*_(228)_ = 1.2, *p* = 0.21). Consistently, they also showed statistical equivalence on the TOST procedure (upper *t*_(228)_ = −2.3, *p* = 0.011; Version A mean = 14.6 ± 2.2; Version B mean = 14.4 ± 2.0). See Supplementary Table S3 for the conversion from FACE Version A and FACE Version B to the global FACE test raw score, on which adjustment based on normative data can be applied (rounded integer values are reported together with the raw scores for the sake of clarity).

We then applied the normative procedure to a sample of 40 PD patients to test the use of this test in clinical practice for the assessment of complex mental state recognition. Our results showed that 28% of PD patients (11 out of 40) had an impaired (*n* = 4) or borderline (*n* = 7) overall performance on the FACE test. Table S4 reports demographic data and FACE test scores with relative adjusted score and ES for each PD patient. The results of partial correlations controlling for MoCA raw score and for multiple comparisons (Table 5) showed significant correlations between FACE test score and social-cognitive measures of emotion recognition (i.e., Ekman 60-Faces Test) and affective ToM (i.e., SET emotion attribution). Further significant correlations were found with attentive-executive functions and action naming.

**Table 5:** Partial correlations, controlling for MoCA raw score, between the FACE test and cognitive measures

	*FACE test*	*FACE test*	
*Digit Backward*	*r*_s_ = 0.176 *p* = 0.303	*SET Global score*	r_s_= 0.443 p = 0.005
*Attentive matrices*	***r***_**s**_ **= 0.551** ***p*** **= 0.0005**	*SET Emotion attribution*	**r**_**s**_ **= 0.493** ***p*** **= 0.001**
*Rey Auditory Verbal Learning delayed recall*	*r*_s_ = 0.181 *p* = 0.290	*SET Intention attribution*	*r*_s_ = 0.417 *p* = 0.008
*Rey-Osterrieth complex figure recall*	*r*_s_ = 0.290 *p* = 0.086	*SET Causal inference*	*r*_s_ = −0.021 *p* = 0.901
*Verbal fluency on phonemic cue*	***r***_**s**_ **= 0.563** ***p*** **= 0.0003**	*Ek60 Global score*	***r***_**s**_ **= 0.464** ***p*** **= 0.0029**
*Stroop time interference effect*	*r*_s_ = −0.273 *p* = 0.113		
*Stroop error interference effect*	*r*_s_ = −0.123 *p* = 0.483		
*Unknown face recognition task*	*r*_s_ = 0.310 *p* = 0.065		
*Line orientation judgment task*	*r*_s_ = 0.181 *p* = 0.291		
*Naming of colored photographs*	*r*_s_ = 0.164 *p* = 0.340		
*Action naming*	***r***_**s**_ **= 0.504** ***p*** **= 0.001**		

Significant results after Bonferroni corrections (*α*_adjusted_ = 0.003) are depicted in bold. *SET*: Story-based Empathy Task, *Ek60*: Ekman 60-Faces Test; *r*_*s*_ = Spearman’s rank correlation coefficient

Finally, ROC analyses showed that an adjusted cut-off score of 26.32 had 78.57% sensitivity and 79.91% specificity in distinguishing PD with socio-cognitive dysfunctions from the normative sample (AUC = 0.821, accuracy = 79.84%). The same cut-off score showed 78.57% sensitivity and 80.77% specificity in distinguishing PD with socio-cognitive dysfunctions from PD without emotion recognition/attribution impairments (AUC = 0.78, accuracy = 80%).

## Discussion

Growing evidence supports the use of social cognitive tasks in the clinical assessment of socio-cognitive deficits in the diagnostic framework of neurodegenerative diseases [16, 19, 30]. However, the clinical availability of these tests is still strongly limited and there is thus an urgent need to develop new standardized tasks to cover the socio-cognitive sub-domains for which no tools are available. Therefore, this study aimed to provide standardization and normative data for a new test of complex mental state recognition through faces (the FACE test). Psychometric properties, normative data, and cut-off scores for the FACE test were provided by analyzing the performances of a sample of 229 healthy Italian participants, taking into account the effect of socio-demographic variables (age, education, and sex). The raw score distribution on the FACE test correlated with age and years of education, resulting in a better performance for younger and more educated participants. This result is consistent with previous studies reporting increased difficulty for older adults in recognizing basic emotions, as well as complex mental states [31], possibly due to age-related physiological modifications of the frontotemporal structures involved in social-cognitive processing [32], rather than to a decline in basic perceptual skills [33]. Furthermore, previous normative studies supported the general role of education in predicting cognitive testing in social cognition tests [25, 28]. On the other hand, no effect of sex was found on test performance. This result is in agreement with previous studies on healthy Italian participants [34, 35], although contrasting results have been also reported [see, for instance, 24].

The usefulness of the FACE test was then demonstrated by applying the correction grids and the equivalent scores to the performance of a sample of 40 PD patients. Difficulties in facial complex-mental-state recognition were found in 28% of our clinical sample, which is in line with recent preliminary evidence suggesting that this clinical population might present relevant socio-emotional impairments in the neuropsychological assessment, even in the early stages of the disease [16, 17]. Moreover, partial correlations showed a significant association between FACE test performance and the results in other social-cognitive tasks of emotion recognition (i.e., Ekman 60-Faces Test) and attribution (i.e., emotion attribution subtask of the Story-based Empathy task), suggesting a possible broad alteration in affective processing in these patients. This hypothesis is further supported by the good accuracy (i.e., 80%) found for the FACE test in distinguishing PD patients with affective socio-cognitive dysfunctions (i.e., emotion recognition and attribution abilities) from PD patients without deficits and healthy controls. At the same time, FACE test performance also correlated with attentive/executive measures. A significant relationship between disorders of social and executive functioning has previously been described and attributed to the involvement of prefrontal cortical areas [36]. Notably, these regions were also found to be involved in action-naming in both healthy [37] and pathological populations [38], suggesting a possible common role of frontal regions affected by PD pathology in causing such a constellation of cognitive impairments. On the other hand, no significant correlations were found with other cognitive domains, supporting overall, in the early stages of the disease, a lack of association of FACE test performance with deficits in semantic or perceptual domains that could affect mental state discrimination or basic facial processing.

Concerning the test construction, the FACE test shares various aspects with another famous test of attribution of complex mental states, the “Reading the Mind in the Eyes Test” (RMET) [39]. Both tasks require participants to choose the term best characterizing the mental states of an actor picture, even though in the RMET only a portion of the face is shown (i.e., eye region). While the RMET has proved to be of considerable value in detecting deficits of mental state representation in neurodevelopmental (e.g., [39]) and neurological disorders, previous studies in PD reported some inconsistencies in the results [15]. This might be at least partially related to the quality of the stimuli used in the original version in terms of image resolution, luminance, and perspective, as well as to the absence of preliminary validation, with the images taken from popular magazine photos. This might significantly affect test validity [40], with possible relevant clinical implications. One example is the cut-off score derived from Italian normative data [34, 35] (cut-off range among studies: 8–16), which, in the best-case scenario, is slightly higher than the score of 13, which represents the minimum score out of 36 for performance significantly above chance [39]. A strength of the FACE test is the inclusion of high-quality stimuli extracted from the McGill Face Database [23], which contains validated pictures representing complex mental states. The 36 final stimuli were selected according to the results of a pilot study investigating the agreement among participants between the facial expression and the complex mental state terms, taking into consideration only those items showing high image and label clarity (see Supplementary Information or details). This procedure also allowed for potential cultural differences between our sample and the original one in the interpretation of facial expressions. Furthermore, the strong correlation with the performance on the Ekman 60-Faces Test [25], another social cognition test assessing emotion recognition from facial expression, supports the potential of the test to measure the underlying construct.

The FACE test is, however, not exempt from limitations. Concerning sample stratification, some co-occurrences of age and education levels happened to be poorly represented (i.e., poorly educated individuals aged <40 years). This should lead to exercising attention when adjusting the raw scores of individuals with these socio-demographic features. Another limitation is the unbalance between the number of FACE stimuli with female and male actors. However, as specified in the “Materials and methods” section, images were preliminary selected based on the results from Schmidtmann et al.’s [23] validation study, showing higher performance for images of the female actor. Finally, the results in the clinical sample and the derived cut-off score should be interpreted while taking into consideration that no patients in the more advanced disease stages were included; thus, caution should be used when applying the cutoff scores proposed here on such individuals. On the other hand, the absence of time limits for a response after stimulus presentation may allow better detection of a deficit in the ability to recognize complex mental states, rather than incorrect or null responses due to a general ideomotor slowdown, which is typical of several neurological conditions, including PD.

In conclusion, growing evidence underlines the importance of introducing social cognitive tests in the neuropsychological assessment of neurodegenerative diseases [16, 19, 30]. The FACE test investigates complex mental state recognition and thus responds to the need for new standardized tasks to cover the socio-cognitive sub-domains for which no validated tools are available. In the spirit of Open Science, test materials, instructions, and cut-off scores are freely available on the web under a Creative Commons license (https://osf.io/dfjcu/?view_only=8e10518adff342c59e77ffe2d7da1903). Therefore, interested neuropsychologists can use them in both clinical and research settings. Moreover, the accessibility of the material may allow authors to replicate results, as well as to translate and adapt the FACE test to specific cultures and languages, and then to proceed with the collection of normative data for populations other than Italian. Finally, the introduction of two short versions of the test responds to clinical and research needs for longitudinal repeated assessments and multiple administrations for monitoring purposing, avoiding the test-retest learning effect. This represents the first test of social cognition in which alternative versions are available, which can be used for both clinical (e.g., monitoring of cognitive profile) and research purposes (e.g., pre-post assessment in clinical trials).

## Supplementary Information

ESM 1: Tables S1–S4 (DOCX 48.2 KB)

## Data Availability

Datasets associated with the current study are available from the corresponding author upon request.
