# Supplementary material for: The FACE test: a new neuropsychological task to assess the recognition of complex mental states from faces
Source: Neurol Sci. 2023 Feb 28;44(7):2339–47. doi: 10.1007/s10072-023-06697-w (PMC10257594; doi:10.1007/s10072-023-06697-w)
Supplement: Supplementary file 1 — Tables S1–S4 (DOCX 48.2 KB) [file 10072_2023_6697_MOESM1_ESM.docx]

**SUPPLEMENTARY INFORMATION**

**Table S1. Summary of the social cognition tasks with available normative data for clinical practice in Italy.**

| **Authors** | **Test** | **Social cognitive facet** |
| --- | --- | --- |
| *Social Perception* |  |  |
| Albonico, Malaspina & Daini [1] | Benton Facial Recognition Test and Cambridge Face Memory Test | Face recognition |
| Dodich et al. [2] | Ekman 60-Faces Test | Basic emotion recognition |
| *Theory of Mind (ToM)* |  |  |
| Dodich et al. [3] | Story-based Empathy Task (SET) | Cognitive and affective ToM |
| Maddaluno et al. [4] and Serafin & Surian [5] | Reading the Mind in the Eyes Test (RMET) | Affective ToM |
| Siciliano et al. [6] | Yoni test from the Edinburgh Cognitive and Beahvioural ALS Screen (ECAS) | Cognitive and affective ToM |
| *Empathy* |  |  |
| Liotti et al. [7] | Brief-Mentalized Affectivity Scale (B-MAS) | Affective empathy |
| Maddaluno et al. [4] | Interpersonal Reactivity Index (IRI) | Cognitive and affective empathy |
| *Decision making* |  |  |
| Maddaluno et al. [4] | Iowa Gambling Test (IGT) | Decision-making |
| *Batteries* |  |  |
| Prior, Marchi & Sartori [8] | i) ToM; ii) emotion attribution; iii) interpretation of social situation; iv) moral judgment | ToM; emotion attribution; interpretation of social situation; moral judgment |

**PILOT STUDY**

A pilot study involving 30 young healthy controls has been performed with the aim to select the most representative stimuli of mental states from the McGill Database [9].

**Stimuli.** Stimuli were extracted from the McGill Face Database [9]. This database consists of high-quality pictures (.jpeg image files with a resolution of 5472 x 3648 pixels; color space profile: sRGB IEC61966-2.1; size of each image: 7.3 MB), taken under controlled conditions in front and side view while two professional English-speaker actors (one male, age 29, and one female, age 23), interpreted 93 expressions of the mental states previously defined in Appendix B of Baron-Cohen et al. [10]. The McGill Face Database is then composed of a total of 372 pictures. For our purpose, only frontal-view pictures were considered. In particular, for each of the 93 mental states, we selected the image of the male/female actor showing the highest expression clarity ratings based on Schmidtmann et al. [9]. All the mental states’ labels were then translated from English to Italian and the obtained labels were re-translated from Italian to English to verify the accuracy of the translation. The process was carried out by an Italian-native professional English translator.

**Pilot study**. 30 healthy young participants (mean age: 25.4 ± 4.32) have been enrolled. For each of the 93 mental states, participants were asked to judge on a 5-point Likert scale (1” corresponds to “*for nothing*”; “*5*” to “*very much*”) the clarity of the image *(CL):* (*e.g.,* the selected picture for <<*TERRIFIED*>> was presented and participants were asked: “How much <<*TERRIFIED*>> does this person seems to you?”). Besides, they were asked to judge the *valence* of the presented pictures by choosing between “*positive*”, “*neutral*” or “*negative*” (*i.e.*, “What is the valence of the image? Remember that we are interested in whether, in your opinion, the emotional state is positive, negative or neutral”) and the *level of arousal* of the presented pictures choosing between “*high*”, “*medium*” or “*low*” (*i.e.*, “What is the level of arousal that the image evokes to you? We are interested in knowing the level of arousal that the person's expression transmits to you. Keep in mind that the level of arousal can be high in both positive and negative emotional states”).

Data on valence and arousal were used in a later stage to define the two short versions. Besides, to define a glossary that could help participants in case of difficulties with mental state labels, participants were also asked to choose, among 3 definitions, the one that best defines each complex mental state. The survey was administered through Qualtrics software (Qualtrics, Provo, UT, USA. <https://www.qualtrics.com>)

**Test construction.** Among the 93 pictures, only those receiving *a CL* rating > 3.5/5 were taken into consideration for test construction. The final set of 36 stimuli was then copied in the center of a slide in a presentation program. Immediately under the picture, four labels representing the target expression and three non-correct alternatives are presented in a row (*Font*: Calibri (Corpo), 18 points, capital letters).

**Short versions.** Two 18-item short versions (i.e., FACE test – Version A and FACE test – Version B) were derived from the 36-item FACE (Table S2). The two sets were matched for CL (Mann-Whitney U = 126, p = 0.26), valence (negative: Mann-Whitney U = 150, p = 0.71, neutral: Mann-Whitney U = 160, p = 0.97 and positive: Mann-Whitney U = 137, p = 0.40) and level of arousal (low: Mann-Whitney U = 161, p = 0.98, neutral: Mann-Whitney U = 153, p = 0.77 and high: Mann-Whitney U = 149 p = 0.69). To facilitate the use of the short versions, R package *Equate* [11, 12] has been used for observed-score linking and equating under single-group using circle-arc functions. Converted scores from short- to long-version are reported in Table S3 for both FACE test – Version A and FACE test – Version B.

**Table S2. List of the stimuli for** **Version A and B of the FACE test.** For each stimulus, the target and alternative responses are shown.

| **FACE test – Version A** | | | | **FACE test – Version B** | | | |
| --- | --- | --- | --- | --- | --- | --- | --- |
| **Target** | **Alternative 1** | **Alternative 2** | **Alternative 3** | **Target** | **Alternative 1** | **Alternative 2** | **Alternative 3** |
| **RALLEGRATO**  (*Amused*) | DESIDEROSO  (*Eager*) | INCURIOSITO  (*Intrigued*) | MINACCIOSO  (*Threatening*) | **SHOCKATO**  (*Aghast*) | SPREZZANTE  (*Hateful*) | DUBBIOSO  (*Doubtful*) | SODDISFATTO  (*Satisfied*) |
| **TERRORIZZATO**  (*Terrified*) | SPREZZANTE  (*Hateful*) | IMBARAZZATO  (*Embarrassed*) | SOLLEVATO  (*Relieved*) | **DIVERTITO**  (*Entertained*) | AFFETTUOSO  (*Affectionate*) | RIFLESSIVO  (*Reflective*) | SOSPETTOSO  (*Suspicious*) |
| **CONTEMPLATIVO**  (*Contemplative*) | AFFASCINATO  (*Fascinated*) | SPIRITOSO  (*Joking*) | COLPEVOLE  (*Guilty*) | **INDECISO**  (*Indecisive*) | COLPEVOLE  (*Guilty*) | SPAVENTATO  (*Fearful*) | AFFABILE  (*Friendly*) |
| **SUPPLICHEVOLE**  (*Imploring*) | MINACCIOSO  (*Threatening*) | SCETTICO  (*Skeptical*) | AFFETTUOSO  (*Affectionate*) | **SOSPETTOSO**  (*Suspicious*) | TURBATO  (*Uneasy*) | SCONCERTATO  (*Flustered*) | DESIDEROSO  (*Eager*) |
| **AFFABILE**  (*Friendly*) | CONVINTO  (*Convinced*) | ENTUSIASTA  (*Enthused*) | PENSIEROSO  (*Thoughtful*) | **APPAGATO**  (*Contented*) | DIVERTITO  (*Entertained*) | ASSORTO  (*Preoccupied*) | CONFUSO  (*Confused*) |
| **PERPLESSO**  (*Perplexed*) | IMBARAZZATO  (*Embarrassed*) | ATTERRITO  (*Panicked*) | RASSICURANTE  (*Reassuring*) | **SCETTICO**  (*Skeptical*) | INSISTENTE  (*Insisting*) | DISORIENTATO  (*Baffled*) | SOGNANTE  (*Fantasizing*) |
| **INORRIDITO**  (*Horrified*) | ACCUSATORIO  (*Accusing*) | ESITANTE  (*Tentative*) | SCHERZOSO  (*Playful*) | **MINACCIOSO**  (*Threatening*) | INDIGNATO  (*Resentful*) | INDIFFERENTE  (*Indifferent*) | SCHERZOSO  (*Playful*) |
| **SODDISFATTO**  (*Satisfied*) | SPIRITOSO  (*Joking*) | ASSORTO  (*Preoccupied*) | ACCUSATORIO  (*Accusing*) | **SOGNANTE**  (*Fantasizing*) | RILASSATO  (*Relaxed*) | ENTUSIASTA  (*Enthused*) | IMBARAZZATO  (*Embarrassed*) |
| **SPAVENTATO**  (*Fearful*) | IRRITATO  (*Irritated*) | SCETTICO  (*Skeptical*) | DESIDEROSO  (*Eager*) | **CONFUSO**  (*Confused*) | INSOLENTE  (*Defiant*) | SERIO  (*Earnest*) | INCORAGGIANTE  (*Encouraging*) |
| **SARCASTICO**  (*Sarcastic*) | INDIGNATO  (*Resentful*) | INDIFFERENTE  (*Indifferent*) | INCORAGGIANTE  (*Encouraging*) | **RIFLESSIVO**  (*Reflective*) | INCURIOSITO  (*Intrigued*) | APPAGATO  (*Contented*) | SECCATO  (*Annoyed*) |
| **SPIRITOSO**  (*Joking*) | RICONOSCENTE  (*Grateful*) | AFFASCINATO  (*Fascinated*) | RAMMARICATO  (*Regretful*) | **ENTUSIASTA**  (*Enthused*) | DESIDEROSO  (*Eager*) | ASSORTO  (*Preoccupied*) | PREOCCUPATO  (*Worried*) |
| **INTERDETTO**  (*Puzzled*) | INSISTENTE  (*Insisting*) | TERRORIZZATO  (*Terrified*) | RILASSATO  (*Relaxed*) | **SCONTENTO**  (*Disappointed*) | INCREDULO  (*Incredulous*) | SPREZZANTE  (*Hateful*) | CONVINTO  (*Convinced*) |
| **ESITANTE**  (*Tentative*) | CURIOSO  (*Curious*) | INORRIDITO  (*Horrified*) | AFFABILE  (*Friendly*) | **SERIO**  (*Earnest*) | INVIDIOSO  (*Jealous*) | SUPPLICHEVOLE  (*Imploring*) | AFFASCINATO  (*Fascinated*) |
| **SBALORDITO**  (*Bewildered*) | SUPPLICHEVOLE  (*Imploring*) | DIFFIDENTE  (*Distrustful*) | SODDISFATTO  (*Satisfied*) | **PENSIEROSO**  (*Thoughtful*) | INCREDULO  (*Incredulous*) | SBALORDITO  (*Bewildered*) | AFFABILE  (*Friendly*) |
| **DOMINANTE**  (*Dominant*) | DISPIACIUTO  (*Apologetic*) | DEMORALIZZATO  (*Dispirited*) | DIVERTITO  (*Entertained*) | **ALLARMATO**  (*Alarmed*) | DISPIACIUTO  (*Apologetic*) | INVIDIOSO  (*Jealous*) | SICURO  (*Confident*) |
| **SCHERZOSO**  (*Playful*) | SICURO  (*Confident*) | ASSORTO  (*Preoccupied*) | SHOCKATO  (*Aghast*) | **SICURO**  (*Confident*) | ENTUSIASTA  (*Enthused*) | RILASSATO  (*Relaxed*) | DISPIACIUTO  (*Apologetic*) |
| **RILASSATO**  (*Relaxed*) | FERMO  (*Assertive*) | SPIRITOSO  (*Joking*) | INCREDULO  (*Incredulous*) | **SOLLEVATO**  (*Relieved*) | SICURO  (*Confident*) | AFFASCINATO  (*Fascinated*) | AGITATO  (*Anxious*) |
| **INFELICE**  (*Depressed*) | TESO  (*Nervous*) | INSOLENTE  (*Defiant*) | CONFORTANTE  (*Comforting*) | **DIFFIDENTE**  (Distrustful) | INDECISO  (*Indecisive*) | SARCASTICO  (*Sarcastic*) | INCURIOSITO  (*Intrigued*) |

**Table S3.** **Score conversion from FACE test – Version A and FACE test – Version B to global FACE test score.**

| *FACE test*  *Version A* | *Global FACE test*  *raw score* | *Global FACE test score*  *(rounded integer values)* | *FACE test*  *Version B* | *Global FACE test*  *raw score* | *Global FACE test score*  *(rounded integer values)* |
| --- | --- | --- | --- | --- | --- |
| 1 | 1.9 | 2 | 1 | 2.1 | 2 |
| 2 | 3.9 | 4 | 2 | 4.1 | 4 |
| 3 | 5.8 | 6 | 3 | 6.2 | 6 |
| 4 | 7.8 | 8 | 4 | 8.2 | 8 |
| 5 | 9.8 | 10 | 5 | 10.2 | 10 |
| 6 | 11.7 | 12 | 6 | 12.2 | 12 |
| 7 | 13.7 | 14 | 7 | 14.3 | 14 |
| 8 | 15.7 | 16 | 8 | 16.3 | 16 |
| 9 | 17.7 | 18 | 9 | 18.3 | 18 |
| 10 | 19.7 | 20 | 10 | 20.3 | 20 |
| 11 | 21.7 | 22 | 11 | 22.2 | 22 |
| 12 | 23.7 | 24 | 12 | 24.2 | 24 |
| 13 | 25.8 | 26 | 13 | 26.2 | 26 |
| 14 | 27.8 | 28 | 14 | 28.2 | 28 |
| 15 | 29.8 | 30 | 15 | 30.2 | 30 |
| 16 | 31.9 | 32 | 16 | 32.1 | 32 |
| 17 | 33.9 | 34 | 17 | 34.1 | 34 |
| 18 | 36.0 | 36 | 18 | 36.0 | 36 |

**Table S4. PD demographic data and FACE test scores with relative adjusted and equivalent score.** Pathological (ES=0) and borderline (ES=1) are depicted in bold and italics, respectively.

| Patient | *Demographic data* | | | *FACE test scores* | | |
| --- | --- | --- | --- | --- | --- | --- |
|  | *Age* | *Education* | *Sex* | *Raw Score* | *Adjusted Score* | *Equivalent Score* |
| 1 | 64.93 | 13 | M | 30 | 30.83 | 4 |
| 2 | 65.61 | 8 | F | 25 | 27.16 | 2 |
| 3 | 60.84 | 13 | F | 33 | 33.53 | 4 |
| 4 | 77.06 | 12 | F | 28 | 29.94 | 4 |
| *5* | *80.46* | *12* | *F* | *21* | *23.18* | *1* |
| 6 | 65.98 | 17 | M | 31 | 31.05 | 4 |
| 7 | 68.19 | 17 | M | 28 | 28.21 | 3 |
| 8 | 69.30 | 10 | M | 24 | 25.87 | 2 |
| 9 | 76.10 | 5 | M | 26 | 29.89 | 4 |
| 10 | 78.11 | 13 | M | 28 | 29.78 | 4 |
| 11 | 68.82 | 11 | M | 30 | 31.58 | 4 |
| 12 | 73.67 | 13 | M | 30 | 31.46 | 4 |
| 13 | 62.84 | 8 | F | 29 | 30.96 | 4 |
| **14** | **71.39** | **5** | **M** | **17** | **20.55** | **0** |
| 15 | 71.47 | 17 | M | 29 | 29.44 | 3 |
| 16 | 70.48 | 8 | M | 29 | 31.51 | 4 |
| 17 | 61.14 | 18 | F | 30 | 29.50 | 4 |
| 18 | 73.95 | 17 | M | 31 | 31.62 | 4 |
| 19 | 78.46 | 13 | M | 23 | 24.80 | 2 |
| **20** | **75.01** | **23** | **M** | **15** | **14.59** | **0** |
| 21 | 73.93 | 13 | M | 25 | 26.48 | 2 |
| 22 | 67.31 | 8 | F | 31 | 33.28 | 4 |
| **23** | **70.47** | **10** | **M** | **16** | **17.96** | **0** |
| 24 | 73.75 | 8 | F | 26 | 28.74 | 3 |
| 25 | 71.91 | 17 | M | 25 | 25.48 | 2 |
| 26 | 71.72 | 8 | F | 27 | 29.60 | 4 |
| 27 | 66.49 | 11 | F | 30 | 31.41 | 4 |
| 28 | 60.77 | 13 | M | 29 | 29.52 | 4 |
| *29* | *65.66* | *13* | *F* | *22* | *22.88* | *1* |
| 30 | 72.19 | 8 | F | 27 | 29.63 | 4 |
| *31* | *82.17* | *8* | *F* | *21* | *24.35* | *1* |
| *32* | *71.05* | *12* | *M* | *23* | *24.50* | *1* |
| 33 | 64.39 | 8 | F | 25 | 27.07 | 2 |
| **34** | **68.44** | **13** | **M** | **21** | **22.08** | **0** |
| 35 | 73.96 | 8 | M | 23 | 25.76 | 2 |
| 36 | 67.91 | 8 | F | 24 | 26.32 | 2 |
| *37* | *72.31* | *10* | *M* | *22* | *24.09* | *1* |
| *38* | *66.44* | *5* | *M* | *20* | *23.19* | *1* |
| 39 | 65.79 | 10 | M | 26 | 27.62 | 3 |
| *40* | *73.81* | *13* | *M* | *22* | *23.47* | *1* |

**REFERENCES**

1. Albonico A, Malaspina M, Daini R (2017) Italian normative data and validation of two neuropsychological tests of face recognition: Benton Facial Recognition Test and Cambridge Face Memory Test. Neurol Sci 38:1637–1643. https://doi.org/10.1007/s10072-017-3030-6

2. Dodich A, Cerami C, Canessa N, et al (2014) Emotion recognition from facial expressions: A normative study of the Ekman 60-Faces Test in the Italian population. Neurol Sci 35:1015–1021. https://doi.org/10.1007/s10072-014-1631-x

3. Dodich A, Cerami C, Canessa N, et al (2015) A novel task assessing intention and emotion attribution: Italian standardization and normative data of the Story-based Empathy Task. Neurol Sci 36:1907–1912. https://doi.org/10.1007/s10072-015-2281-3

4. Maddaluno O, Aiello EN, Roncoroni C, et al (2022) The Reading the Mind in the Eyes Test, Iowa Gambling Task and Interpersonal Reactivity Index: Normative Data in an Italian Population Sample. Arch Clin Neuropsychol. https://doi.org/10.1093/arclin/acab100

5. Serafin M, Surian L (2004) Il test degli occhi: uno strumento per valutare la" Teoria della Mente". G Ital di Psicol 31:839–862. https://doi.org/10.1421/18849

6. Siciliano M, Trojano L, Trojsi F, et al (2017) Edinburgh Cognitive and Behavioural ALS Screen (ECAS)-Italian version: regression based norms and equivalent scores. Neurol Sci 38:1059–1068. https://doi.org/10.1007/s10072-017-2919-4

7. Liotti M, Spitoni GF, Lingiardi V, et al (2021) Mentalized affectivity in a nutshell: Validation of the Italian version of the Brief-Mentalized Affectivity Scale (B-MAS). PLoS One 16:. https://doi.org/10.1371/journal.pone.0260678

8. Prior M, Marchi S, Sartori G (2003) Cognizione sociale e comportamento, volume I: Uno strumento per la misurazione. Domeneghini Editore, Padova

9. Schmidtmann G, Jennings BJ, Sandra DA, et al (2019) The McGill Face Database: validation and insights into the recognition of facial expressions of complex mental states. Perception 49:310–329. https://doi.org/10.1101/586453

10. Baron-Cohen S, Wheelwright S, Hill J, et al (2001) The “Reading the Mind in the Eyes” Test revised version: A study with normal adults, and adults with Asperger syndrome or high-functioning autism. J Child Psychol Psychiatry Allied Discip 42:241–251. https://doi.org/10.1017/S0021963001006643

11. Albano AD (2016) equate: An R package for observed-score linking and equating. J Stat Softw 74:1–36. https://doi.org/10.18637/jss.v074.i08

12. Albano AD (2016) equate: Observed-Score Linking and Equating in R. Appl Psychol Meas 40:361–362. https://doi.org/10.1177/0146621615620553
